# Low T3 Syndrome as a Predictor of Poor Prognosis in Patients With Pyogenic Liver Abscess

**DOI:** 10.3389/fendo.2019.00541

**Published:** 2019-08-06

**Authors:** Jing Xu, Liang Wang

**Affiliations:** Diabetes Center and Department of Endocrinology, The Second Affiliated Hospital and Yuying Children's Hospital of Wenzhou Medical University, Wenzhou, China

**Keywords:** low T3 syndrome, pyogenic liver abscess, poor prognosis, mortality, thyroid

## Abstract

**Aim:** There is an association between the low triiodothyronine (T3) state and the poor prognosis for severe acute conditions. However, the correlation between thyroid dysfunction and pyogenic liver abscess (PLA) is unclear. This study aims to figure out how low T3 syndrome is related to the poor prognosis in PLA patients as well as estimate the serum T3 predictive value.

**Methods:** The study consecutively enrolled 240 PLA patients in total with a 3 month followed-up period, and defined low T3 syndrome as low T3 level together with non-thyroid disease. Researchers implemented multivariate logistic regression analyses, univariate analysis, as well as receiver-operating characteristic (ROC) curve analysis.

**Results:** Patients with low T3 syndrome had a higher mortality rate (14.3 vs. 2.0%), acute hepatic failure (6.8 vs. 1.0%), and septic shock (12.1 vs. 3.0%) than patients with normal levels of T3 (all *P* < 0.05). Low T3 syndrome served as an independent predictor of death [odds ratio (OR) = 5.03, 95% of confidence interval (CI) = 1.09–23.05], and all adverse outcomes [odds ratio (OR) = 3.63, 95% of confidence interval (CI) = 1.84–7.17] following the adjustment of potential confounders in the logistic model. T3 had the largest area under the ROC curve (AUC) than T4, FT3, FT4, and TSH in death prediction (AUC = 0.901, cut-off value = 0.70 nmol/L, *P* < 0.01), and all adverse outcomes (AUC = 0.743, cutoff value = 0.83 nmol/L, *P* < 0.01).

**Conclusions:** It seems that low T3 syndrome can predict the prognosis of PLA in clinical practice in future.

## Introduction

As a disease rarely reported, pyogenic liver abscess (PLA) is able to seriously threat patients' life. In North America, the incidence rate reaches 2.3:10,000, and that in Taiwai Area reaches 275.4:100,100 (1, 2). Enhanced diagnostic approach together with proper treatment method help to reduce the mortality of PLA to a large extent in the range of 10–40% (3, 4). However, there remains some diagnostic issues and therapeutic problems (5, 6). Prognostic markers should be identified to provide patients with more positive and timely resuscitation and treatment method should be planed for PLA patients in the future for a better prognosis in the emergency room (2).

Low level of T3 syndrome (also known as sick euthyroid syndrome or non-thyroidal disease syndrome), lowered peripheral concentrations of T3 together with normal level of thyroid stimulating hormone (TSH) represent that the thyroid hormone metabolism of critical ill patients has changed (7). A growing evidence has indicted that clinic outcomes in patients with low T3 syndrome were associated with multiple trauma (8), myocardial infarction and heart failure (9), cerebrovascular diseases (10), liver cirrhosis (11), and chronic kidney disease (12). With regard to sever infection, Jinliang Liu et al. concluded that low T3 syndrome could lead to 30 day mortality and ICU admission in hospitalized community-acquired pneumonia (13). Based on the study of Luo et al., low T3 syndrome can predict sepsis patients' poor prognosis (14). However, there was no previous literature that paid attention to PLA patients with low T3 syndrome as far as we know.

The study aims at studying how low T3 syndrome is related with PLA, as well as the prognostic value exhibited by low T3 syndrome in the identification of adverse outcomes in PLA patients.

## Materials and Methods

### Study Design

Researchers carried out a retrospective review of the medical records of all PLA patients who were admitted from Jan 2014 to Dec 2016 to the Second Affiliated Hospital and Yuying Children's Hospital of Wenzhou Medical University. A total of 240 hospitalized PLA patients were retrieved through the International Classification of Diseases (Revision 9) search agency database. This study has obtained the approval from the Ethics Committee of the Second Affiliated Hospital of Wenzhou Medical University (No. LCKY2017-01) and has obtained the written informed consent of all subjects following the Declaration of Helsinki.

### Study Population

Inclusion criteria: Patients who have no less than one lesion in the imaging of liver such as ultrasound (US) or computed tomography (CT), or whose regression of radiological abnormalities is complete or the blood pus culture is positive following the antimicrobial therapy. Exclusion criteria: Patients who have amoebic liver abscesses and cirrhosis.

### Data Collection and Outcome Measurements

Researchers implemented a retrospective review of the clinical records of patients, that paid attention to patients' demographic characteristics like the age and gender; comorbid diseases such as diabetes mellitus and, hypertension; clinical features like body weight, height, and temperature as well as systolic and diastolic blood pressure (BP); Laboratory findings involved white blood cell (WBC) count, neutrophil (N) count, red blood cell (RBC), hemoglobin (Hb), platelet (PLT) count, C-reactive protein (CRP), procalcitonin, prothrombin time (PT), platelet (PLT), activated partial thromboplastin time (APTT), high-density lipoprotein cholesterol (HDL-c), total cholesterol (TC), triglyceride (TG), low-density lipoprotein cholesterol (LDL-c), albumin, serum creatinine, uric acid, alanine aminotransferase (ALT), aspartate transaminase (AST), total bilirubin, klebsiella pneumoniae (KP) infection, T3, T4, FT3, FT4, and TSH. All these findings were provided by individual medical records on admission based on the prespecified definitions.

Blood samples for thyroid function tests were obtained within 24 h of hospital admission. An AIA 600 system (Tosho Corporation) with full automation helped to measure the thyroid hormones at the hospital laboratory. The reference values for the laboratory were: TSH, 0.49–4.91 mIU/L; T4, 69.96–152.52 nmol/L; T3, 1.01–2.48 nmol/L; FT3, 3.28–6.47 pmol/L; and FT4, 7.64–16.03 pmol/L. Patients with low T3 syndrome refer to those whose TSH level is normal and whose T3 level is smaller than the lower limit of reference interval (T3 < 1.01 nmol/L) (15).

Hypertension was defined as the recent use of antihypertensive drugs, a systolic blood pressure of ≥140 mmHg, and/or a diastolic blood pressure of ≥90 mmHg. Diabetes mellitus was defined as the recent use of antidiabetic drugs, a fasting glucose value of ≥7.0 mmol/L, a casual glucose value of ≥11.1 mmol/L. Anemia was defined as hemoglobin <13 g/dl in men and <12 g/dl in women. The body mass index (BMI) was calculated as weight (Kg) divided by height squared (m^2^). The Geriatric Nutritional Risk Index (GNRI), which is as nutritional index, was calculated as follows: GNRI = 14.89 × serum albumin (g/dl) + 41.7 × %body weight. %body weight = measured body weight (Kg)/[22 × square of height (m)].

We estimated the thyroid's secretory capacity (SPINA-GT), referred to as thyroid output of thyroid capacity, sum activity of peripheral deiodinases (SPINA-GD), which reflects the maximum stimulated activity of step-up deiodination, and thyrotropic function as well as the thyrotroph thyroid hormone resistance index (TTSI) (16).

The authors recorded the treatments of each patient according to microbiological detection and imaging findings, including antibiotics alone, antibiotics + surgery, or antibiotics + percutaneous catheter drainage (PCD), and adverse outcomes, such as empyema, mortality, metastatic infection (referring to distal infection of which the bacterium as PLA culture are the same); septic shock (the definition and treatment follow the Surviving Sepsis Campaign criteria) (17); acute respiratory failure (referring to patients' requirement for the mechanical ventilation); acute hepatic failure (referring to the growth of serious acute liver injury accompanied by coagulopathy and encephalopathy); hospitalized acute myocardial infarction (part of acute myocardial infarction); acute renal failure in hospitalization (the serum creatinine increased by 0.5 mg/dL from baseline); as well as upper gastrointestinal (UGI) bleeding (referring to hemorrhage related to pressure under endoscopic observation, with bright red blood). In addition, the mortality rate of patients for 3 months was observed in the study.

### Statistical Analysis

Researchers expressed continuous data as median [interquartile range] or means ± standard deviations, and carried out the Mann-Whitney U test or an independent *t*-test. They also expressed categorical data as frequencies (%) and implemented Fisher's exact or chi-squared test. Spearman's correlation was adopted to estimate the correlation between T3, FT3, SPINA-GD, markers of inflammation, and liver function. The possible predictors were decided with univariate logistic regression analysis, and these predictors were found to cause death or all adverse outcomes. We selected each parameter, and *P*-value was <0.05 based on the initial univariate analysis results. A forward stepwise variable selection step helped to finally determine the gender, age, creatinine, and PT used in the minimal model of multivariable logistic regression analysis. In order to classify the density and all adverse outcomes of these PLA patients in the future, we plotted a receiver operating characteristic (ROC) curve. The IBM SPSS Statistics 20.0, statistical computer programs (SPSS Inc., USA), helped to analyze these data and *P* < 0.05 was deemed significant.

## Results

### Baseline Characteristics

A total of 240 patients suffering PLA were enrolled, the mean age was 67.6 ± 10.7 years, 60.8% were male, and the mean serum T3 concentration was 0.94 ± 0.44 nmol/L. Besides, 56.2% patients suffered low T3 syndrome with PLA based on diagnosis. The basic characteristics of survivor and non-survivors were showed in Table 1. Compared with survivor patients, non-survivor patients were older, with lower BMI, RBC, hemoglobin, albumin, TC and HDL-c, but higher procalcitonin, PT levels, as well as detection rate of non-klebsiella pneumoniae. Parallel to this, non-survivors exhibited obviously lower levels of serum T4, T3, and FT3 in comparison with survivors (all *P* < 0.01). Rather, the two groups exhibited no obvious difference in the level of serum FT4. Also, the serum level of thyroid hormone helped to divide participants into two groups, group with low T3 syndrome (*n* = 135), and group without low T3 syndrome (*n* = 105), as shown in Table 2. Compared with patients without low T3 syndrome, patients with low T3 syndrome were older, with significantly higher temperature, WBC, CRP, total bilirubin, and PT levels. Group with low T3 syndrome exhibited greatly lower albumin, RBC, SPINA-GT, and SPINA-GD levels compared with group with normal T3 syndrome.

**Table 1:** The comparison of the clinical and liver imaging between survivors and non-survivors groups.

	**Overall (*n* = 240)**	**survivors (*n* = 219)**	**Non-survivors (*n* = 21)**	***P***
Age, years	67.6 ± 10.7	66.8 ± 13.6	76.6 ± 11.2	0.002
Male	146 (60.8)	135 (61.6)	11 (52.4)	0.635
Co-morbidity				
Hypertension	67 (27.9)	61 (27.9)	6 (28.6)	1.000
Diabetes	114 (47.5)	106 (48.4)	8 (38.1)	0.494
BMI, Kg/m^2^	23.3 ± 2.9	23.4 ± 2.9	22.0 ± 2.8	0.099
SBP, mmHg	125 ± 20	125 ± 21	130 ± 16	0.257
DBP, mmHg	71 ± 11	71 ± 11	73 ± 11	0.442
Temperature, °C	39.3 ± 0.8	39.4 ± 0.8	39.1 ± 0.9	0.360
White blood cell, 10^9^/L	12.2 ± 6.0	12.1 ± 5.9	13.3 ± 7.1	0.351
Neutrophil count, 10^9^/L	10.17 ± 5.89	10.06 ± 5.85	11.41 ± 6.39	0.146
Temperature, °C	39.3 ± 0.8	39.4 ± 0.8	39.1 ± 0.9	0.360
Red blood cell, 10^12^/L	4.0 ± 0.6	4.0 ± 0.6	3.6 ± 0.5	0.006
Hemoglobin, g/L	118.9 ± 18.3	120.1 ± 18.0	105.9 ± 17.0	0.001
Platelet count, 10^9^/L	222.9 ± 133.9	226.1 ± 127.2	188.5 ± 193.8	0.309
C-reactive protein, mg/L	90.3 ± 68.8	91.3 ± 70.7	76.4 ± 33.5	0.584
Procalcitonin, pg/ml	13.7 ± 23.6	12.1 ± 22.1	27.4 ± 31.9	0.044
Creatinine, umol/L	72.7 ± 54.1	71.9 ± 55.1	81.9 ± 40.9	0.489
Total bilirubin, mg/L	16.2 ± 14.5	16.0 ± 14.3	18.6 ± 16.8	0.535
ALT, U/L	71 ± 72	73 ± 73	51 ± 54	0.145
AST, U/L	68 ± 86	65 ± 78	99 ± 142	0.134
Albumin, g/L	30.8 ± 6.7	31.3 ± 6.7	25.3 ± 4.6	<0.001
Uric acid, umol/L	241 ± 240	239 ± 91	265 ± 134	0.291
TC, mmol/L	3.5 ± 1.0	3.6 ± 1.0	3.1 ± 0.8	0.049
TG, mmol/L	1.5 ± 0.9	1.5 ± 0.9	1.5 ± 0.8	0.793
HDL-C, mmol/L	0.6 ± 0.4	0.7 ± 0.4	0.4 ± 0.3	0.011
LDL-C, mmol/L	2.1 ± 0.8	2.1 ± 0.8	1.7 ± 0.7	0.074
PT, s	14.9 ± 1.6	14.8 ± 1.6	15.8 ± 1.5	0.007
APTT, s	41.8 ± 6.0	41.8 ± 5.8	41.2 ± 7.2	0.615
GNRI	89.5 ± 11.7	90.4 ± 11.4	79.7 ± 10.3	<0.001
T4, nmol/L	97.5 ± 31.4	98.9 ± 31.0	81.6 ± 32.6	0.009
T3, nmol/L	0.94 ± 0.44	0.99 ± 0.42	0.40 ± 0.21	<0.001
FT4, pmol/L	12.94 ± 3.78	12.92 ± 3.43	13.10 ± 6.64	0.999
FT3, pmol/L	3.59 ± 0.89	3.68 ± 0.86	2.61 ± 0.45	<0.001
TSH, mIU/L	1.87 ± 1.50	1.93 ± 1.55	1.31 ± 0.74	0.050
KP infection	180 (74.8)	169 (77.7)	11 (53.8)	0.047
Abscess features				
Solitary lesion	189 (78.8)	175 (80.3)	14 (65.0)	0.147
Mean size of abscess, cm	6.3 ± 2.8	6.3 ± 2.8	6.0 ± 2.9	0.617

*Values are expressed as mean ± SD or number (%); P < 0.05 was deemed significant (comparison between survivors and non-survivors group); ALT, alanine aminotransferase; AST, aspartate Transaminase; TC, total cholesterol; TG, triglyceride; HDL-c, high-density lipoprotein cholesterol; LDL-C, low-density lipoprotein cholesterol; KP, klebsiella pneumoniae; PT, prothrombin time; APTT, activated partial thromboplastin time; GNRI, Geriatric Nutritional Risk Index*.

**Table 2:** The comparison of the clinical and liver imaging between low T3 syndrome and without low T3 syndrome groups.

	**Overall (*n* = 240)**	**Low T3 syndrome (*n* = 135)**	**Without low T3 syndrome (*n* = 105)**	***P***
Age, years	67.6 ± 10.7	69.8 ± 12.4	64.7 ± 14.8	0.004
Male	146 (60.8)	83 (61.5)	63 (60.0)	0.894
Co-morbidity				
Hypertension	67 (27.9)	37 (27.4)	30 (28.6)	0.885
Diabetes	114 (47.5)	66 (48.9)	48 (45.7)	0.696
BMI, Kg/m^2^	23.3 ± 2.9	23.1 ± 2.9	23.5 ± 3.0	0.274
SBP, mmHg	125 ± 20	126 ± 21	123 ± 19	0.275
DBP, mmHg	71 ± 11	71 ± 11	71 ± 12	0.897
Temperature, °C	39.3 ± 0.8	39.5 ± 0.8	39.1 ± 0.8	0.041
White blood cell, 10^9^/L	12.2 ± 6.0	12.8 ± 6.5	11.5 ± 5.3	0.047
Neutrophil count, 10^9^/L	10.17 ± 5.89	10.68 ± 6.29	9.52 ± 5.31	0.131
Red blood cell, 10^12^/L	4.0 ± 0.6	3.9 ± 0.6	4.1 ± 0.6	0.049
Hemoglobin, g/L	118.9 ± 18.3	118.2 ± 17.1	119.8 ± 19.7	0.497
Platelet count, 10^9^/L	222.9 ± 133.9	221.8 ± 137.8	224.3 ± 129.2	0.887
C-reactive protein, mg/L	90.3 ± 68.8	102.9 ± 63.1	75.5 ± 72.8	0.044
Procalcitonin, pg/ml	13.7 ± 23.6	16.2 ± 27.3	9.3 ± 14.2	0.151
Creatinnine, umol/L	72.7 ± 54.1	75.9 ± 66.7	68.6 ± 31.1	0.302
Total bilirubin, mg/L	16.2 ± 14.5	17.7 ± 16.8	14.2 ± 10.6	0.045
ALT, U/L	71 ± 72	73 ± 73	69 ± 72	0.652
AST, U/L	68 ± 86	73 ± 86	63 ± 85	0.373
Albumin, g/L	30.8 ± 6.7	29.8 ± 6.8	32.1 ± 6.5	0.011
Uric acid, umol/L	241 ± 240	239 ± 92	244 ± 99	0.648
PT, s	14.9 ± 1.6	15.0 ± 1.7	14.6 ± 1.5	0.048
APTT, s	41.8 ± 6.0	41.7 ± 5.8	41.8 ± 6.1	0.897
GNRI	89.5 ± 11.7	87.7 ± 12.0	91.8 ± 11.0	0.006
SPINA-GT, pmol/s	9.6 ± 7.1	7.7 ± 4.7	12.0 ± 8.8	<0.001
SPINA-GD, nmol/s	29.5 ± 14.5	21.7 ± 7.6	39.4 ± 15.4	<0.001
TTSI, mIU/L	146.0 ± 113.7	153.2 ± 107.9	136.8 ± 120.7	0.268
KP infection	180 (74.8)	95 (70.6)	85 (82.1)	0.249
Abscess features				
Solitary lesion	189 (78.8)	108 (80.2)	81 (76.6)	0.623
Mean size of abscess, cm	6.3 ± 2.8	6.0 ± 2.6	6.6 ± 2.9	0.134

*Values are expressed as mean ± SD or number (%); P < 0.05 was deemed significant (comparison between with and without low T3 syndrome group); ALT, alanine aminotransferase; AST, aspartate Transaminase; KP, klebsiella pneumoniae; PT, prothrombin time; APTT, activated partial thromboplastin time; GNRI, Geriatric Nutritional Risk Index; SPINA-GT, thyroid's secretory capacity; SPINA-GD, sum activity of peripheral step-up deiodinases; TTSI, thyrotroph thyroid hormone sensitivity index*.

### Spearman’s Correlation

**Figure 1:**
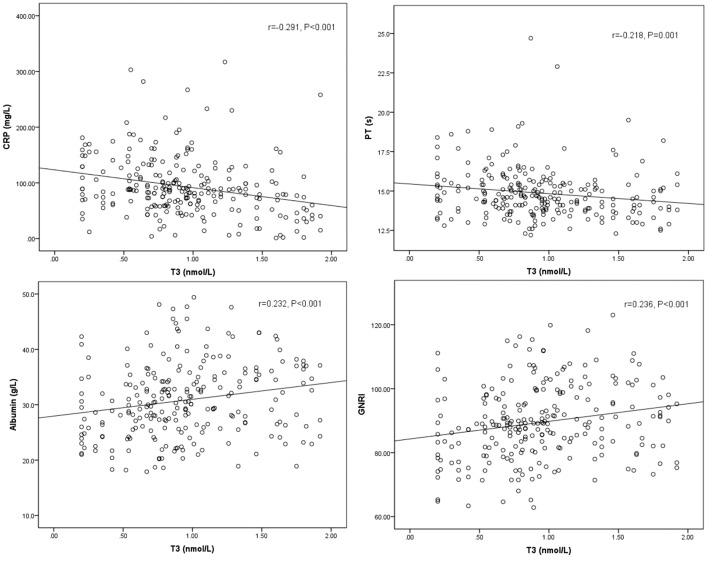
Scatter diagrams showing correlation between T3, CRP, PT, albumin, and GNRI. T3 had a negative association with CRP (*r* = −0.291, *P* < 0.001), PT (*r* = −0.218, *P* = 0.001), and positively correlated with albumin (*r* = 0.232, *P* < 0.001), and GNRI (*r* = 0.236, *P* < 0.001).

Spearman's correlation between T3, FT3, SPINA-GD, markers of inflammation, and liver function is shown in Table 3 and Figure 1. T3 had a negative association with CRP and PT. FT3 was negatively correlated with PT. SPINA-GD was significantly and negatively correlated with CRP, total bilirubin and PT. T3, FT3, and SPINA-GD were positively correlated with albumin and GNRI.

**Table 3:** Correlation analysis between T3, FT3, SPINA-GD, markers of inflammation and liver function.

	**T3**		**FT3**		**SPINA-GD**	
	***r***	***p***	***r***	***p***	***r***	***P***
White blood cell	−0.107	0.099	−0.104	0.109	−0.106	0.101
C-reactive protein	−0.291	< 0.001	−0.086	0.390	−0.309	0.002
Procalcitonin	−0.162	0.096	−0.110	0.258	−0.099	0.310
Temperature	−0.066	0.326	−0.065	0.334	−0.062	0.352
Albumin	0.232	< 0.001	0.176	0.006	0.233	< 0.001
Total bilirubin	−0.115	0.076	−0.074	0.254	−0.128	0.048
PT	−0.218	0.001	−0.197	0.002	−0.209	0.001
GNRI	0.236	< 0.001	0.202	0.002	0.229	< 0.001

*SPINA-GD, sum activity of peripheral step-up deiodinases; PT, prothrombin time; GNRI, Geriatric Nutritional Risk Index*.

### Clinical Outcomes

Four (1.7%) patients underwent antibiotics plus surgery, 110 (45.8%) took antibiotics alone, and 126 (52.5%) received antibiotics + PCD. Group with low T3 syndrome presented significantly higher treatment of antibiotics + PCD compared with group without low T3 syndrome (*P* = 0.049). The low T3 syndrome group exhibited obviously higher hospitalization expense (*P* = 0.045) than normal T3 group. The incidence rate (*P* = 0.001) of all adverse outcomes of patients in group with low T3 syndrome increased by 24.6 to 41.8% from the 17.2% in normal T3 group, which involved the higher mortality rate (14.3 vs. 2.0%), acute hepatic failure (6.8 vs. 1.0%) and septic shock (12.1 vs. 3.0%) (Table 4). The low T3 syndrome had a number needed to harm (NNH) of 4 for all adverse outcome, and had an NNH of 8 for death. The beta error value of all adverse outcome was 1.1%, and the beta error value of death was 6.4%.

**Table 4:** The comparison of the treatment and clinical outcome between low T3 syndrome and without low T3 syndrome groups.

	**Overall (*n* = 240)**	**Low T3 syndrome (*n* = 135)**	**Without low T3 syndrome (*n* = 105)**	***P***
Percutaneous drainage	126 (52.5%)	77 (57.0)	49 (46.7)	0.049
Operation	4 (1.7)	2 (1.5)	2 (1.9)	0.466
Hospital length of stay, days	17.6 ± 9.2	18.0 ± 9.5	16.9 ± 8.7	0.356
Hospitalization expenses, 10^4^ CNY	3.2 ± 2.5	3.5 ± 2.6	2.9 ± 2.2	0.045
Adverse outcomes	75 (31.3)	57 (41.8)	18 (17.2)	0.001
Mortality	21 (9.1)	19 (14.3)	2 (2.0)	0.001
Metastatic infection	33 (13.6)	21 (15.3)	12 (11.3)	0.439
Acute renal failure	9 (3.9)	8 (6,0)	1 (1.0)	0.082
Acute hepatic failure	10 (4.3)	9 (6.8)	1 (1.0)	0.047
Acute respiratory failure	11 (4.7)	7 (5.2)	4 (4.0)	0.763
Acute myocardial infarction	8 (3.4)	7 (5.3)	1 (1.0)	0.143
UGI bleeding	18 (7.8)	14 (10.4)	4 (4.1)	0.087
Empyema	38 (15.9)	24 (17.9)	14 (13.3)	0.369
Septic shock	20 (8.2)	17 (12.1)	3 (3.0)	0.015

*Values are expressed as mean ± SD or number (%); P < 0.05 was deemed significant (comparison between with and without low T3 syndrome group); UGI bleeding, upper gastrointestinal bleeding*.

### Univariate and Multivariate Logistic Regression Analysis

Univariate analysis demonstrated the association between age ≥65, PLT <125, PT >14.8 s, GNRI <90 and low T3 syndrome and density; however, age ≥65, anemia, PLT <125, diabetes, hypertension, GNRI <90, and low T3 syndrome were associated with the poor prognosis. Based on multivariate analysis, low T3 syndrome, PLT <125 and GNRI <90 were significant predictors for the density; low T3 syndrome, anemia, PLT <125 and diabetes were significant predictors for the poor prognosis in PLA patients (Tables 5, 6).

**Table 5:** Univariate and minimal model of multivariable logistic regression for risk factors associated with mortality.

	**Univariate analysis**	**Multivariate analysis**
**Variable**	**Odd ratio (95%CI)**	**Odd ratio (95%CI)**
Age≥65 years	4.23 (1.48–12.06)^*^	
Male	0.74 (0.29–1.87)	
Anemia^a^	3.56 (1.31–9.62)^*^	
PLT <125, 10^9^/L	3.51 (1.38–8.94)^*^	2.88 (1.01–8.20)^*^
PT>14.8 s	3.23 (1.19–8.72)^*^	
Percutaneous drainage	0.80 (0.32–2.02)	
Size>6 cm	1.92 (0.64–5.75)	
Creatinine>1.3 mg/dL	2.50 (0.65–9.55)	
Diabetes	0.71 (0.28–1.80)	
Hypertension	0.84 (0.29–2.43)	
GNRI <90	7.45 (1.69–32.91)^*^	4.37 (1.04–20.34)^*^
Low T3 syndrome	7.59 (1.72–33.54)^*^	5.03 (1.09–23.25)^*^

a *Hemoglobin <13 g/dL in men, <12 g/dL in women; CI, confidence interval*.

* *P < 0.05*.

**Table 6:** Univariate and minimal model multivariable logistic regression for risk factors associated with adverse outcomes.

	**Univariate analysis**	**Multivariate analysis**
**Variable**	**Odd ratio (95%CI)**	**Odd ratio (95%CI)**
Age≥65 years	2.14 (1.22–3.76)^*^	
Male	0.79 (0.45–1.39)	
Anemia^a^	1.81 (1.03–3.17)^*^	1.99 (1.06–3.73)^*^
PLT <125, 10^9^/L	3.11 (1.67–5.78)^*^	3.82 (1.90–7.71)^*^
PT>14.8 s	1.72 (0.99–3.01)	
Percutaneous drainage	0.71 (0.41–1.23)	
Size>6 cm	1.02 (0.48–2.15)	
Creatinine>1.3 mg/dL	1.80 (0.95–3.39)	
Diabetes	1.74 (1.00–3.05)	1.99 (1.06–3.75)^*^
Hypertension	1.82 (1.00–3.31)^*^	
GNRI <90	2.77 (1.51–5.09)^*^	
Low T3 syndrome	3.46 (1.85–6.47)^*^	3.63 (1.84–7.17)^*^

a *Hemoglobin <13g/dL in men, <12g/dL in women; CI, confidence interval*.

* *P < 0.05*.

### Prognostic Value of Low T3 Syndrome

**Figure 2:**
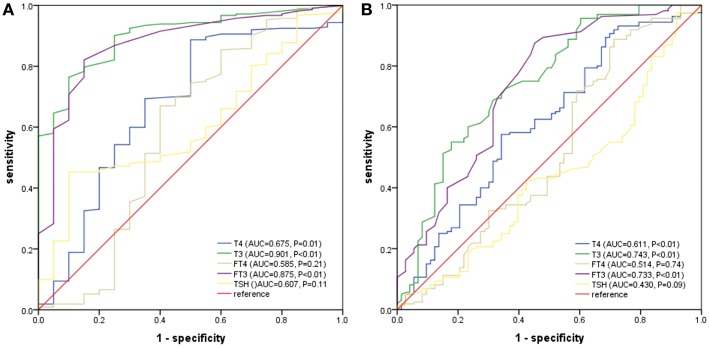
**(A)** ROC analysis of T4, T3, FT3, FT4, and TSH to death among PLA patients. **(B)** ROC analysis of T4, T3, FT3, FT4, and TSH to adverse outcomes among PLA patients. T3 had the largest area under the ROC curves, and it had statistical significance (0.901 for density and 0.743 for all the adverse outcomes). The optimal cut-off value of T3 for predicted death was 0.70 nmol/L and that of all adverse outcome was 0.83 nmol/L.

For a comparison between the mortality and all poor prognosis in terms of the predictability among PLA patients, we plotted the ROC curves for T4, T3, FT3, FT4, and TSH, as shown in Figures 2A,B. We can clearly see that T3 had the largest area under the ROC curves, and it had statistical significance (0.901 for density and 0.743 for all the adverse outcomes). The optimal cut-off value of T3 for predicted death was 0.70 nmol/L and that of all adverse outcome was 0.83 nmol/L.

## Discussion

The association between low concentration of thyroid hormone, especially low concentration of serum T3, and the prognosis of serious non-thyroid diseases, including sepsis and respiratory failure, has been reported. As far as we know, this is the first study which proves that low T3 syndrome can be used to well-predict the adverse prognosis of PLA patients. Whether low T3 syndrome is related with severe infectious disease in pathophysiology fails to be fully explained, however, the thyroid hormone profile, a simple parameter which is easily measured may be used to predict the prognosis of PLA in clinical practice in the future as a good candidate.

In the past 20 years, the correlation between low T3 syndromes and critically ill patients has been reported, and the prevalence of severe trauma patients has reached 90.0% (8), of patients with end-stage renal disease has reached 78.6% (12), of acute stroke patients has reached 56.6% (18), of patients suffering respiratory failure reached 53.1% (19), of patients with community-acquired pneumonia has reached 31.8%(13), and of heart disease patients has reached 30.2% (9). Among 240 PLA patients enrolled in our study, 135 (56.2%) presented low T3 syndrome at first admission, significantly higher than many severe illnesses. Therefore, it is necessary to fully evaluate and manage the thyroid hormone dysfunction in PLA patient care.

As for baseline characteristic, we found that patients with low T3 syndrome had higher temperature, WBC, CRP, total bilirubin, and PT levels, but lower RBC and albumin than patients without low T3 syndrome. As shown in Table 3, additionally, we showed the association between T3 level and C-reactive protein (*r* = −0.291, *P* < 0.001), albumin (*r* = 0.232, *P* < 0.001), PT (*r* = −0.218, *P* = 0.001), and GNRI (*r* = 0.236, *P* < 0.001). Consistent with the findings in the study in a set of laboratory data, Fan et al. found that serum T3 was positively related to the hemoglobin and the protein-energy nutrition (serum albumin), but negatively related to the inflammation (CRP) (12). Considering the multiple risk index associated with adverse outcomes, the low T3 syndrome suggests that it is necessary for doctors to take into account more careful resuscitation as well as timely and proper treatment methods.

According to previous epidemiological studies, low T3 syndrome could be used to independently predict the poor survival in acute cerebrovascular disorders (10), chronic heart failure (20), end-stage renal disease (21), and hemodialysis (22). Nevertheless, whether low concentration of T3 serum can directly affect the prognosis and outcome of PLA patients remains speculative. Actually, researchers have found the relation between low T3 level and the growth of sepsis (14), respiratory failure (19) as well as multi-organ dysfunction syndrome (23), and low T3 level also serves as the primary complication, and common death cause of PLA patients. Consistent with this, we found in our study that PLA in low T3 syndrome patients had a worse prognoses (including mortality, acute hepatic failure, and septic shock) than patients with normal T3 level. We also demonstrated that low T3 syndrome was an independent risk factor for both mortality and adverse outcomes.

The plot showed that T3 levels had larger AUCs in comparison to T4, FT3, FT4, and TSH in mortality and adverse outcomes prediction (0.901 for mortality and 0.743 for all adverse outcomes). According to many previous studies, PLA has many other prognostic values (2, 6, 24), such as older age, low level of albumin, and hemoglobin, increased BUN and serum creatinine, polymicrobial infection, biliary liver abscesses, multiple abscesses, concomitant malignancy, and pleural effusions. The study also found that GNRI was greatly related to the poor prognosis in PLA patients (25). By now, we have not figured out the most proper biomarker to predict the liver abscess. Nevertheless, compared with other factors, low T3 syndrome as a biomarker boosts a huge advantage, due to its simplicity, economic efficiency, easy availability, and minimal patient involvement.

For acute illness, the occurrence of low T3 syndrome can be explained by changes in thyroid hormone binding, peripheral thyroid hormone uptake as well as expression activity of the type-1 deiodinases (D1), and type-3 deiodinases (D3). As patients with acute critical illnesses usually experience concomitant fasting, the decreased thyroid hormone availability may reflect an adaptive attempt to reduce energy expenditure and, thus, appearings to be beneficial. In addition, the increased D3 activity could optimize the bacterial killing capacity of neutrophilic granulocytes. Sterling and Eyer extended the classical paradigm of homeostasis with the theory of allostasis. Type1 allostatic load will occur, if energy demands exceed the sum of energy intake and the amount of energy that can be mobilized from stores (26). For example, thyroid hormone metabolism may change in muscle due to the pathogenesis of myopathy related to long-term ventilator dependence (27).

Specific to acute and severe disease such as PLA, mechanical connections could be bidirectional, and multifactorial, like D3 levels, inflammation, as well as protein consumption. A large number of inflammatory cytokines, like tumor necrosis factor (TNF)-α, interleukin (IL)-1, and IL-6, can restrain 1 5′-deiodinase expression, accordingly causing T4 to converse to T3, as a result, the T3 production will be decreased (28). The association between these cytokines with the poor prognosis in PLA was also demonstrated (29). Insufficient nutrition and high metabolic consumption in PLA may also serve as the hypothetical mediators in the development of low T3 syndrome for reducing the energy expenditure (25). In addition, D3, viewed as the major thyroid hormone inactivating enzyme, was shown to be highly expressed in infiltrating neutrophilic granulocytes during bacterial infections, and that may lead to low T3 levels during an acute infection (30).

Despite potential promise, people always debate about if thyroid hormone administration is useful for treating low T3 syndrome. Pappa et al. proposed the necessity for further validating if thyroxine supplementation is effective in clinical research which is well-designed (31), whereas De Groot et al. set forth that it is necessary to appropriately treat low T3 syndrome, which manifests hypothalamic-pituitary dysfunction, using replacement therapies (32). Despite the controversy about the effectiveness of exogenous T3 replacement in treating low T3 syndrome, it is suggested to perform an intervention study to investigate if normalized T3 value is able to enhance the PLA survival, which may help to better discriminate the causal relationship of low T3 syndrome in PLA.

The research suffered three limitations. (1), the research was a retrospective single-center study. (2), the mortality rate of PLA patients during hospitalization was much smaller (21 case of death). (3), the endocrine-based test results may be associated with the sampling time.

## Conclusion

In conclusion, low T3 syndrome was associated with the poor prognosis in PLA patients, and it was an independent prognostic factor for all adverse outcomes and mortality. Serum T3 predictive value used to predict adverse outcomes was superior to that of T4, FT3, FT4, and TSH. Thyroid hormone levels are highly reproducible and show easy measurement in all diagnostic labs. It is necessary to perform longitudinal studies with longer follow-up period and larger sample size in the future to explain the effect of low T3 syndrome as a marker of critical illness, a metabolic adaptation, or a predictor of the poor prognosis in PLA patients.

## Data Availability

The datasets generated for this study are available on request to the corresponding author.

## Ethics Statement

This study was carried out in accordance with the recommendations of Human Body Research of guidelines, Hospital Institutional Review Board of committee with written informed consent from all subjects. All subjects gave written informed consent in accordance with the Declaration of Helsinki. This study has obtained the approval from the Ethics Committee of the Second Affiliated Hospital of Wenzhou Medical University (No. LCKY2017-01).

## Author Contributions

LW study concept and design, preparation, review, and approval of manuscript. JX data collection and interpretation, preparation, review, and approval of manuscript.

### Conflict of Interest Statement

The authors declare that the research was conducted in the absence of any commercial or financial relationships that could be construed as a potential conflict of interest.

## Abbreviations

PLA: Pyogenic liver abscess
TSH: thyroid-stimulating hormone
BMI: body mass index
HbA1c: glycosylated hemoglobin
TC: total cholesterol
TG: triglyceride
HDL-c: High density lipoprotein cholesterol
LDL-c: Low density lipoprotein cholesterol
GNRI: Geriatric Nutritional Risk Index PLA patients.
